# Editorial: Long-non coding RNAs in renal cell carcinoma

**DOI:** 10.3389/fonc.2022.997525

**Published:** 2022-08-16

**Authors:** Zongping Wang, Song Wang, An Zhao

**Affiliations:** ^1^ Department of Urology, Cancer Hospital of University of Chinese Academy of Sciences (Zhejiang Cancer Hospital), Hangzhou, China; ^2^ Institute of Cancer and Basic Medicine (ICBM), Chinese Academy of Sciences, Hangzhou, China; ^3^ Experimental Research Center, Cancer Hospital of University of Chinese Academy of Sciences (Zhejiang Cancer Hospital), Hangzhou, China

**Keywords:** long non-coding RNA, lncRNA, renal cell carcinoma, genitourinary cancers, oncology

Renal cell carcinoma (RCC) is one of the most common urological malignancies and is known for its complex genomic heterogeneity and natural drug resistance (1). Identifying the key regulatory mechanisms of RCC and developing biomarker-based diagnosis and treatment strategies for RCC have been expected in clinical practice (2, 3). Among the many kinds of tumor biomarkers, long non-coding RNAs (lncRNAs) has recently attracted considerable attention due to its various of functions in cellular processes (4, 5). Increasing evidence supports the role of lncRNA in tumor development, metastasis and drug resistance in RCC (6, 7). Therefore, lncRNAs have been suggested as biomarkers and topics in novel diagnostic and therapeutic strategies for RCC.

In this current topic, an overview of lncRNAs in RCC is provided through 4 original research papers by 23 authors, and these studies expand our understanding of the important roles of lncRNAs in the progression of RCC (8–11). Notably, Tan et al. reported the overexpressed lncRNA DUXAP9 was associated with poorer overall survival and progression-free survival in clear cell RCC (ccRCC). DUXAP9 knockdown can inhibit RCC cell proliferation, motility capacities and reverse epithelial-mesenchymal transition (EMT), whereas overexpression of DUXAP9 promoted RCC cells proliferation and motility capacities *in vitro* and induced EMT. Interesting, RNA immunoprecipitation and RNA stability assays showed that DUXAP9 was methylated at N6-adenosine and binds to IGF2BP2, which increases its stability. DUXAP9 activate PI3K/AKT pathway and Snail expression in RCC cells. DUXAP9 may be useful as a prognostic marker and/or therapeutic target in localized ccRCC.

The identification of RCC-associated lncRNAs using multi-omics and systems biology has been reported by serval groups. Chen et al. identified 4 key hypoxia-related lncRNAs (COMETT, EMX2OS, AC026462.3, and HAGLR) based on TCGA-KIRC datasets, and verified their relative expression *via* the qRT-PCR method, then they construct signature and nomogram to predict the prognosis of ccRCC patients. They also found that the 4-lncRNAs based-risk score was remarkably related to the infiltration levels of 6 tumor immune cells, they proposed that this study may be useful for medical decision-making and targeted therapy. Su et al. constructed a ceRNA network of key genes that are significantly associated with the distant metastasis and prognosis of patients with ccRCC. The distant metastasis-related lncRNAs were used to construct a risk score model through the univariate, least absolute shrinkage selection operator (LASSO), and multivariate Cox regression analyses, and the patients were divided into high- and low-risk groups according to the median of the risk score. The Kaplan–Meier survival analysis demonstrated that mortality was significantly higher in the high-risk group than in the low-risk group. qRT-PCR in the tissues and cells of ccRCC verified the high-expression level of three lncRNAs. Gene set enrichment analysis revealed that the lncRNA prognostic signature was mainly enriched in autophagy- and immune-related pathways. Interestingly, Fang et al. identified the genome instability-related lncRNAs (GInLncRNAs) and their clinical significances in RCC, based on the mutation data and lncRNA expression data on the TCGA database, they determined 11 GInLncRNAs to construct a prognostic model, and found that this model was significantly associated with the RCC patients’ overall survival, their study provided theoretical support for the exploration of the formation and development of RCC.

In summary, this Research Topic provides novel insights into the regulatory network of lncRNAs in RCC, and these findings reveal the potential applications of lncRNAs in the diagnosis and therapy of RCC. We hereby appreciate all the authors for contributing to this Research Topic.

## Author contributions

AZ drafted the editorial. ZW and SW edited the manuscript. All authors contributed to this work and gave approval to the final version.

## Conflict of interest

The authors declare that the research was conducted in the absence of any commercial or financial relationships that could be construed as a potential conflict of interest.

## Publisher’s note

All claims expressed in this article are solely those of the authors and do not necessarily represent those of their affiliated organizations, or those of the publisher, the editors and the reviewers. Any product that may be evaluated in this article, or claim that may be made by its manufacturer, is not guaranteed or endorsed by the publisher.
